# The continuing evolution of ownership

**DOI:** 10.1371/journal.pone.0211871

**Published:** 2019-02-12

**Authors:** Tilman Hartley

**Affiliations:** School of Sociology, Politics, and International Studies, University of Bristol, Bristol, United Kingdom; Shandong University of Science and Technology, CHINA

## Abstract

The evolution in animals of a first possession convention, in which individuals retain what they are the first to acquire, has often been taken as a foundation for the evolution of human ownership institutions. However, among humans, individuals actually only seldom retain an item they have acquired from the environment, instead typically transferring what they possess to other members of the community, to those in command, or to those who hold a contractual title. This paper presents a novel game-theoretic model of the evolution of ownership institutions as rules governing resource transfers. Integrating existing findings, the model contributes a new perspective on the emergence of communal transfers among hominin large game hunters around 200,000 years ago, of command ownership among sedentary humans in the millennia prior to the transition to agriculture, and of titled property ownership around 5,500 years ago. Since today’s property institutions motivate transfers through the promise of future returns, the analysis presented here suggests that these institutions may be placed under considerable pressure should resources become significantly constrained.

## Introduction

**Fig 1:**
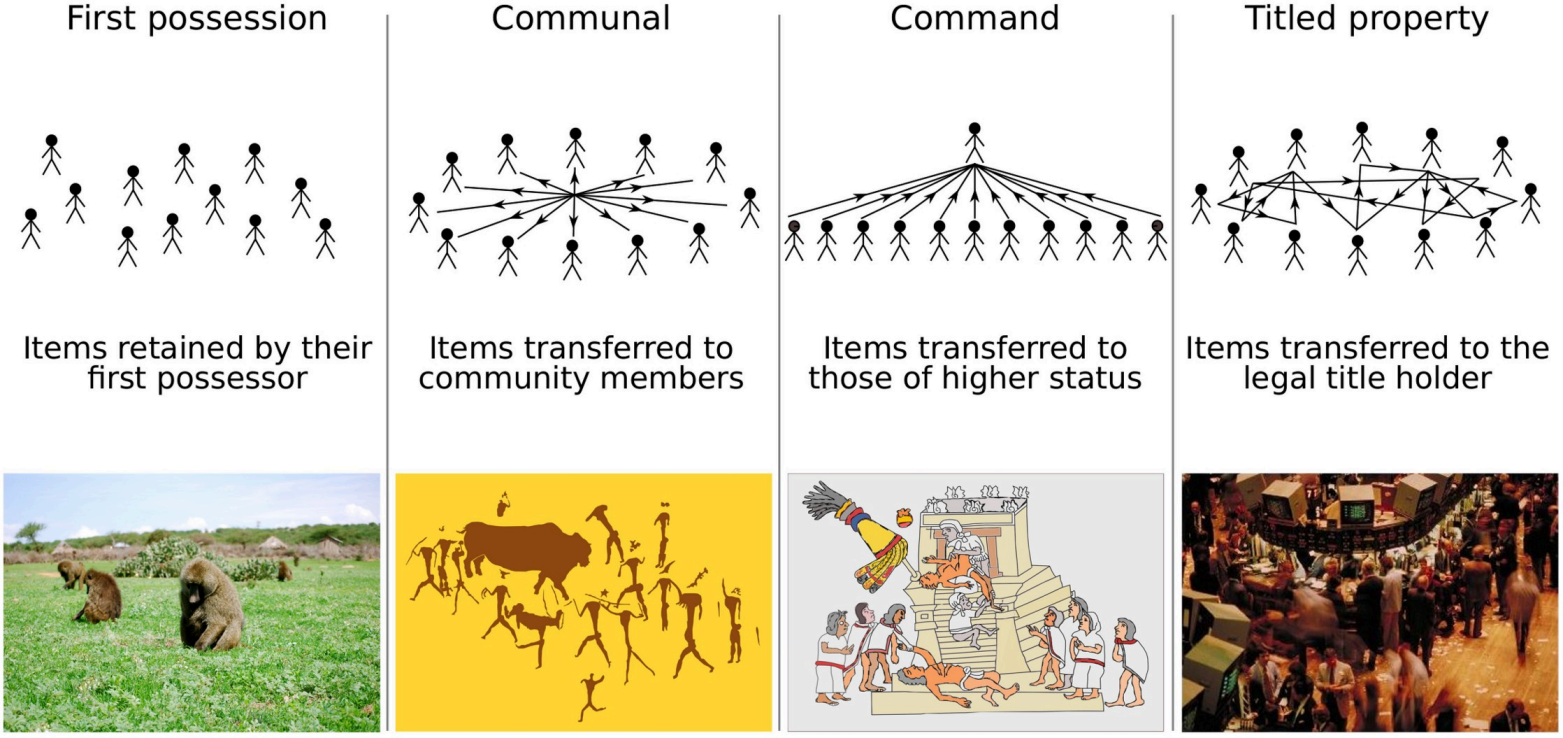
Four ownership institutions governing the retention or transfer of resource items. Foraged foods tend to be retained by their first possessor, whilst under communal ownership norms items are transferred to others in the group; under command ownership items are transferred to those of higher status, whilst under titled property items are transferred to the holder of legal title. ‘A troop of olive baboons’ photo reprinted under a CC BY license with permission from Amanda Lea, original copyright 2009; cave art after San rock painting in the Drakensberg Mountains; Aztec ritual after an extract from the Codex Magliabechiano; New York Stock Exchange image is in the public domain.

One of the earliest applications of evolutionary game theory was to model how nonhuman animals signalling an intent to defend territory prevents wasteful conflict within groups [1, 2], and a ‘first possession’ convention is sometimes taken as the basis for human ownership institutions [3–7]. However, among humans, resource items are usually not retained by their first possessor but are transferred to others. These transfers are governed by different ownership rules (see Fig 1): under the ‘communal’ ownership norms that evolved among hunters of large game resources are transferred to other group members; under the ‘command’ ownership typical of hierarchical sedentary societies resources are transferred to those of higher status; and under ‘titled property’ ownership resources are transferred to the holder of a legal title to those resources.

The need for a clearer understanding of the mechanisms by which a society’s institutions change alongside its resource base has been noted by researchers across disciplines [8–11]. In evolutionary terms, the general question addressed in this paper can be stated as: Why do different ownership institutions survive to govern different resource types? Stated more specifically: Why did ‘communal’ ownership norms evolve among hunters of large game? Why did persistent ‘command’ ownership tend to survive among sedentary societies? And in what conditions have contractual ‘titled property’ ownership institutions historically survived?

In the next section, the model is outlined and the results and predictions are set out. This provides a heuristic for an examination of the existing empirical evidence for the evolution of communal, command, and titled property institutions. This is followed by a ‘Discussion’ section in which the main similarities and differences between this and other models, as well as the model’s main limitations, are discussed. A short ‘Conclusion’ summarises the contributions that this paper makes to the literature. At the end of the paper, a ‘Methods’ section sets out the model in full.

## An evolutionary game theory model: Results and predictions

The model builds on the intuition that each individual can either obtain some resource item from the environment or can demand some resource item from someone else; and that an individual who has obtained a resource from the environment can either surrender it upon demand or can resist such demands. This yields three discrete strategies: a Demander demands resources from others and fights if necessary; a Resister obtains a resource from the environment and fights to defend it if necessary; and a Transferrer obtains a resource from the environment but surrenders it if demanded. Five parameters determine the strategy payoffs (see Table 1).

**Table 1:** Payoff matrix for the Demander-Resister-Transferrer game.

	Demand	Resist	Transfer
Demand	0	*Pv* − *f*	*v*
Resist	(1 − *P*)*v* − *f* − *c*	*v* − *c*	*v* − *c*
Transfer	*b* − *c*	*v* − *c*	*v* − *c*

Payoffs are to the row player (on the left). Consuming a resource item increases an individual’s fitness (by a value *v*), but there is a fitness reducing cost to obtaining it from the environment (*c*). There are costs to both Demanders and Resisters when they fight (*f* for both). Transferrers may receive some benefit when they make a transfer (*b*). The proportion of conflicts won by Demanders when they fight against Resisters is the fifth and final variable of the model (*P*).

**Fig 2:**
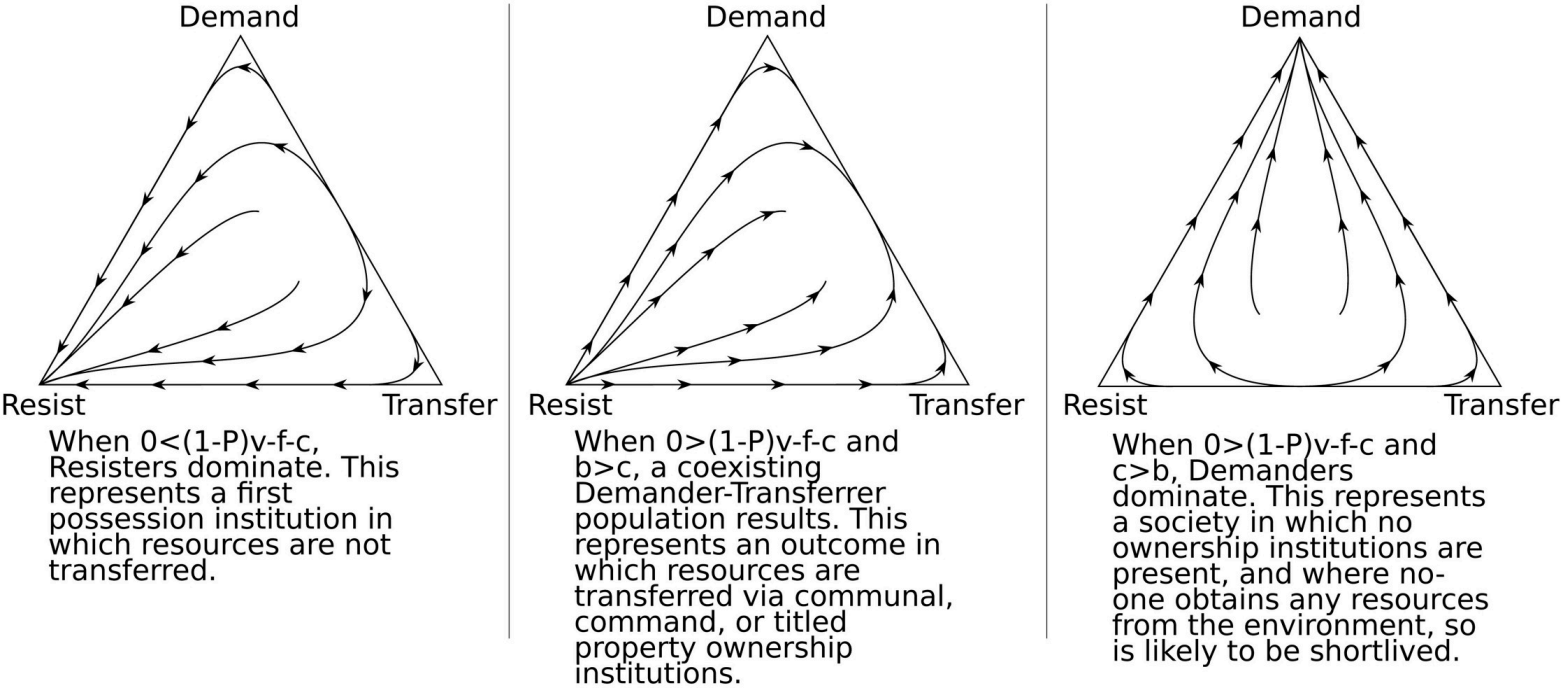
The three game outcomes. Three evolutionary stable outcomes can result: a population dominated by Resisters; one where Demanders and Transferrers co-exist; or one where Demanders dominate.

Though some forms of institutionalism take institutions to be exogenous, the explicitly evolutionary approach taken here interprets institutions as selected from a diversity of possibilities, and evolving as the result of a change in a given population of individuals [12, 13]. Individuals adopt different strategies with regard to the acquisition, retention, and transfer of items, and population change occurs as individuals playing the more fitness enhancing strategies increase as a proportion of the population [14]. Different values for the five variables therefore lead to different mixes of strategies in the population. An ‘evolutionary stable’ outcome occurs when a given set of parameter values results in a population playing one or more strategies can prevent ‘invasion’ by those playing a different strategy. The model yields three sets of conditions in which three evolutionary stable outcomes result (see Fig 2).

As summarised in Fig 2, the model results yield predictions for the conditions in which ‘communal’, ‘command’, and ‘titled property’ institutions will tend to survive in a population. These predictions provide a heuristic for an examination, in the next section, of the circumstances in which different ownership institutions have evolved and survived. First possession is predicted to survive as long as 0 < (1 − *P*)*v* − *f* − *c*, which could be rewritten as *f* + *c* < (1 − *P*)*v*; that is, first possession survives as long as the net increase in fitness that accrues to Resisters after their losses to Demanders ((1 − *P*)*v*) is greater than the fitness costs of obtaining and defending (*c* + *f*) the resource. Correspondingly, a shift away from first possession is predicted when 0 < (1 − *P*)*v* − *f* − *c* no longer holds; if, alongside this condition, the fitness costs of obtaining a resource is greater than the benefits of transferring, so that *c* > *b*, then the population is predicted to be dominated by Demanders, but probably only quite briefly since no resources are being acquired by anyone in such a population.

The population will not become dominated by Demanders, however, as long as there are sufficient benefits to making transfers, relative to the costs of resource acquisition, to maintain *b* > *c*; in that case, a mixed Demander-Transferrer population results, corresponding to a communal, command, or titled property institution. The transition to communal ownership, then, is predicted to occur when there are increases in the costs of fighting between group members (*f*), which might occur with the development of better hunting tools which can also be used as weapons, and when there are sufficient increases in the fitness benefits of transferring to others (*b*) in relation to the costs of acquiring resources from the environment (*c*), such as when sustaining other group members provides sufficient benefits in the form of collective defence or a reduction in the unpredictability of the food supply. It is predicted that communal ownership institutions will no longer be maintained if it becomes more costly to hunt, for example as large game declines, or if there are fewer benefits of sustaining other group members, as then the inequality *b* > *c* may no longer hold; a Demand dominated population may briefly occur.

Alternatively, command ownership is predicted to survive where 0 > (1 − *P*)*v* − *f* − *c* and *b* > *c* which may occur where, for example, those demanding resources are sufficiently likely to win resource conflicts as this reduces the net fitness of Resisters, and where the benefits of group defence remains high relative to cost of resource acquisition, for example if conflict between groups escalates so that the defensive benefits of group membership remain high.

Finally, titled property ownership emerges as a means to ensure that individuals receive a benefit from transferring what they have previously acquired, so that the inequality *b* > *c* holds both for those who transfer resources in expectation of receiving a return as well as for those without inherited wealth who can acquire resources for investment. This prevents a return to a first possession institution by ensuring individual benefits to transferring resources, as well as both the incentives and an investment mechanism for a society to obtain increasing amounts of resources.

## The evolution of communal, command, and titled property ownership

Proceeding roughly chronologically, in this section I will examine evidence for the circumstances in which communal, command, and titled property ownership institutions emerged and survived, situating the model in existing debates around these institutional developments.

### Big game hunting and communal ownership

Inferences for the way in which communal ownership may have evolved amongst hominins has been drawn from the behaviour of other social carnivores, of nonhuman primates, and of extant hunter-gatherer groups. About 3.5 million years ago species of canid, felid, and primate social carnivores emerged to fill the ecological niches created by sunnier drier conditions [15]. Since most terrestrial mammalian carnivores are solitary, increased sociality is thought to have evolved as a derived trait where group life increases individual fitness, including through collective defence and, particularly in species that hunt large game, through the acquisition of more resources [15, 16]. However, competition over food is typically sufficiently intense to disrupt grouping behaviour, and most social carnivores are therefore structured by fission-fusion dynamics in which groups break up in times of scarcity and reassemble when food is abundant [17, 18]. Though some species of nonhuman primate share rare or difficult to process foods with weaned offspring, and in some of these species there is also sharing between adults in mating or defensive coalitions, among nonhuman primates systematic sharing between non-kin is rare [19]. More commonly observed among nonhuman primates is an increased toleration for the scrounging or taking of food, particularly when the initial possessor has large fruits or large quantities of meat that it would be difficult to defend against others, though foods of such large package size do not typically constitute a large proportion of their diet [8, 19, 20].

Among human hunter-gatherers, however, active food sharing is ubiquitous [8, 19, 21]. It is thought that widespread sharing beyond kin allows a larger group to be maintained, the benefits of which includes collective defence against predators and a reduction in the unpredictability of the food supply, often called ‘variance reduction’ [8, 22, 23]. In terms of the model, these are fitness enhancing benefits (*b*) of transferring resources to others in the group. Though hominin meat consumption dates to at least 2.5 million years ago [24] and the hunting of small bovids and persistent carnivory from scavenging or hunting to around 2 million years ago [25, 26], the earliest evidence for large animal hunting is much later, following physiological changes that allowed accurate overhead throwing, as well as the capacity for speech which may have allowed greater collective coordination. The development of tools for large game hunting are also thought to have massively increased the costs of fighting (*f*) between group members wielding those tools as weapons [27]. By around 250,000 years ago large game had become a prominent component of human subsistence [25], and around 200,000 years ago the earliest evidence is found for butchery of large animals by a single butcher [28]. These hominin hunters of large game appear to have peaceably taken carcasses back to central places to be divided and shared among other members of the group. This marks a distinction between the behaviour of humans and that of other primates who do not take meat from the kill to provision others who are absent [27, 29, 30]. Among human hunter-gatherers, successful hunters typically neither claim ownership of what they have acquired nor control any aspect of its distribution [8, 19, 21]. For example, among the Netsilik Eskimo twelve of the fourteen portions cut from a seal are distributed to a network of meat-sharing partners deliberately chosen during childhood to be outside the existing close relationships of the hunter [31, 32]; among the !Kung of the Kalahari the distributor of the meat is chosen on the basis that their arrow was the first to hit the animal, but since arrows are regularly exchanged between hunters this is often not the hunter who took the shot, and may even be someone not present at the time [21]; and among the Ache of Paraguay it was long considered taboo for a hunter to eat portions of their kills, and meat distribution is still usually undertaken by an older man in the group [22].

### Sedentism, stratification, and command ownership

Though archaeological evidence suggests sporadic experiments with institutional inequality as long as 29,000 years ago, stratification seems to have become more permanent only much later among more sedentary societies [33]. Theories of the evolution of institutional stratification include narratives of both the beneficial and the coercive aspects of hierarchical societies [34]. The coercive aspects are thought to have become easier to sustain in sedentary societies where some people prevent others from accessing resources [35]; though the value of resources (*v*) may increase relative to the costs of acquiring them from the environment (*c*) and the costs of fighting (*f*), a return to a first possession institution can nevertheless be avoided if those demanding resources are sufficiently likely to win resource conflicts (*P*). Meanwhile, the benefits of some level of stratification might include the creation of role models, the provision of dispute resolution, a division of labour, and the collective punishment of free-riders [36–41]. These benefits, however, do not differ greatly from those of belonging to a mildly stratified hunter-gatherer society, a similarity that perhaps helps explain why the relatively egalitarian institutions of horticulturalists tend to resemble those of hunter-gatherers [42].

Beginning around 15,000 years ago, the archaeological record shows the start of what has become known as the ‘quaternary extinction’, a period in which many species of larger animals became extinct [43–45]. The model suggests that if such a decline leads to it becoming more costly to hunt and there being fewer benefits of sustaining other group members, the benefits of transferring resources may become less that the costs of obtaining them in the first place (*c* > *b*) and so communal ownership institutions may no longer be maintained. Groups may briefly become dominated by those who demand resources from others, or fission as other social carnivores do when competition for food becomes too intense [17, 18] as occurs in a Demand-dominated population in the model. During periods of scarcity, several hunter-gatherer groups who practice no cultivation whatsoever define areas within which individuals forage for low value resources, resources that hunter-gatherers typically do not share beyond immediate family [46, 47]. This shift in behaviour is well documented amongst groups such as those of the American northeast and the!Kung, who seasonally switch from a pattern of communal living in the summer where game is large and plentiful to a system of separate plots in the winter when game is small and when noncultivated plants provide a more important contribution to the diet [47, 48]. Periods of abundance occurred even less frequently for the Shoshone of Nevada, with tribes fissioning into separate multi-family camps and often remaining isolated for years at a time [47, 49]. It may be that a similar process occurred after the decline of large game during the Late Quaternary.

In western Asia, beginning around 12,800 years ago, clusters of small huts each probably housing a nuclear family appear in the archaeological record [50]. This non-agricultural but largely sedentary Natufian culture already shows signs of some social stratification in the form of differences in height, grave goods, and housing, though both sedentism and stratification disappeared during the more variable climatic conditions of the Younger Dryas from around 12,900 to 11,600 years ago [51–53]. With the return of a more stable climate, sedentary settlements returned to western Asia [54]. By around 10,000 years ago, the early occupants of Abu Hureya in Syria lived in sedentary clusters of five to seven small houses, gathering wild rye, wild barley, two kinds of wild wheat and hunting, mostly gazelles; two thousand years later, occupants of the same site harvested domesticated wheat and barley and herded domesticated sheep and goats [32, 55]. Though in western Asia, China, Japan, and northwestern Peru permanent settlements are thought to have preceded cultivation often by millennia, in India, Africa, and north America such settlements may have only emerged after the process of domestication was already well under way [56–58]. Despite this diversity of pathways, across all sites paleobotanic evidence suggests that the rate at which wild plants were domesticated is comparable to the rate at which variation in wild varieties occur, suggesting that domesticates resulted from hundreds or thousands of years of selection pressure by at least seasonally- or semi-sedentary humans [56].

In the light of recent ethnographic and archaeological evidence, the popular theory that domestication was delayed until the creation of an institution of individual land ownership [59–61] is no longer so strongly supported [46]. Though ethnographic examples have sometimes been drawn upon to suggest that a lack of the appropriate ownership institutions may have been an impediment to the transition to agriculture, these examples are of mobile hunter-gatherers such the Hadza of Tanzania, the Batek of Malaysia, the !Kung, and the Hiwi of Venezuela, groups that normally obtain a large proportion of their diet from hunting and who actively maintain communal ownership [46, 61]. Indeed, recent ethnographic studies suggest that the institutions of extant low level horticulturalists far more closely resemble the more egalitarian practices of hunter-gatherers than the institutions of agriculturalists or pastoralists [42], and even among agriculturalists cases can be found in which communal ownership persisted in societies with a high dependence on domesticates, such as the Natchez of the American southwest who managed the entire crop as a communal effort and shared the produce [47].

Though land ownership may not have been a prerequisite for domestication, some level of sedentism has historically allowed wealth, power, and status to be accumulated not only by individuals, but by lineages [62]. This increases the likelihood (represented in the model by *P*) that these powerful elites will win any conflicts with those who acquire resources from the environment, and thus prevents a return to a first possession institution. Even in nonagricultural societies sedentism often coincided with hereditary power, for example in the salmon-rich American Pacific northwest where chiefs commanded tributes and held slaves—though nonagricultural societies never reach the levels of surplus and differences in power achieved in agricultural societies [32, 63–65]. Hereditary rule is often resisted, with societies cycling between periods in which status is hereditary and periods in which such leaders are overthrown [32, 33, 65, 66]. But where such rulers do become established they are often able to command significant resources [35, 67]. Chiefs commanding greater resources and more soldiers are typically able to outcompete those with fewer resources and smaller armies [37, 38, 68]; sometimes one chiefdom would achieve decisive victory over its neighbours, and sometimes one such unified kingdom was able to subordinate neighbouring kingdoms to create an empire [32, 37, 69]. High levels of conflict, evidenced by defensive structures, deadly raids, and the abandonment of affected farmland suggest that, given the alternatives, there would have been significant benefits to group membership (*b*) for even the lowliest member of society [32, 70]. These defensive benefits may provide benefits to group membership even if these individuals receive only just enough to eat to survive: Mesopotamian labour records from the end of the third millennium BC suggest that both coerced labour and unskilled wage labourers received rations at bare subsistence level [71].

### Contracts, growth, and titled property

Whilst a strong literature had suggested that secure ownership affords owners economic incentives and so drives economic growth [60, 72, 73], it has been noted that secure ownership has often been present during historical periods where little such growth occurred [74, 75]. An emerging literature now highlights a particular characteristic of property titles that had been previously neglected: the ability of owners to use their titles to procure loans [76, 77]. This literature now suggests that the use of property titles to procure loans drives economic growth by increasing both the availability of resources for investment and the incentives for seeking returns, particularly by individuals keen to avoid dispossession by default. Though beginning as an essentially extractive practice [78], the creation of titles to future resources does ensure that individuals receive a return on resources they transfer to others, and provides a means for those without inherited wealth to obtain resources for investment (so ensuring that *b* > *c* and a return to first possession is avoided). However, the relationship between property titles and economic growth is also hypothesised to be bidirectional [79, 80]: the wider availability of resources to fund investments provide both the incentives and the means for individuals to increase their income through loans that can generally be repaid during times of resource expansion, but periods without expansion have historically been accompanied by instability caused by social polarisation, and often a return to more directly coercive forms of ownership [81].

Unlike earlier ownership institutions, the evolution of contractual titles to resource items can largely be traced through the written historical record; indeed, writing itself likely first developed in Mesopotamia around 5500 years ago for the purpose of recording such contracts [78, 82, 83]. The imprinted clay used to seal shipments became used to record the goods with which a merchant had been entrusted for sale elsewhere [84] effectively becoming contracts for the delivery of a specific quantity of resources to be transferred at a specified future time [78]. Likely a uniquely Mesopotamian innovation, the interest-bearing loan arose once lenders began to expect a fixed rate of return on these commercial loans [78]. Sumerian rulers [85] and later wealthy private creditors [86] extended the logic of these commercial agreements and began to charge a fixed rate of interest on loans to farmers who pledged themselves and their family to debt bondage in the event of default; labour markets soon emerged as wages likely became the only means by which the indebted could repay their debts and avoid enslavement [71, 78, 86]. By the end of the 3rd millennium BC, the enslavement of farmers was having a negative effect on social cohesion, and in the centuries leading up to 1600 BC rulers periodically proclaimed ‘clean slates’ in which agrarian debts—but not commercial loans—were forgiven and the enslaved allowed to return to their place of origin, reasserting the royal right to agricultural tributes and overriding the claims of private creditors [85]. However, rulers gradually lost their power and by the first millennium BC were no longer able to annul the titles acquired by increasingly wealthy private creditors [33, 78].

Contracts for interest-bearing loans appear in Greece during the eighth century BC, likely due to the influence of Syrian and Phoenician merchants [87]. Lacking a strong central authority with the ability to cancel debts and redistribute property titles, the arrival of interest soon led to dispossession, debt peonage, and popular revolts against the ruling oligarchs in cities across Greece [88]. In Athens, Solon’s 594 BC reforms cancelled all debts and outlawed debt bondage and dependent labour among Athenians, though unlike the Mesopotamian system this was a one-off measure and crises soon recurred [89]. Gradually a cycle of oligarchic rule and revolt gave way to ever more stable political institutions which, though institutionalising the exclusion of women and of slaves, would include all the male citizenry in decision-making [90]. Though repeatedly identified by contemporary observers as a source of social tension, interest charges persisted throughout the Greek classical period [91]. Consistent returns were made possible by an economy growing between about 0.07 and 0.14 percent per year in the period 800-300 BC, coinciding with increases in trade across the Mediterranean and territorial expansion westward: between 750 and 500 BC the area of arable land under Greek control roughly doubled [90].

The early centuries of the Roman Republic, its beginning traditionally dated to 509 BC, were similarly characterised by debt crises. Successive legislation was passed to limit interest and wealth concentration [92–95]. A key innovation was to permit interest charges where the lender suffers a loss such as through wear and tear, but to prohibit usurious contracts that charged for merely using a thing where no such loss occurs; charges on consumption loans, specifically, were classified as usury since the repayment of a quantity of food or drink equal to that consumed by the borrower was considered full repayment of what had been borrowed [96, 97]. The Roman success in overcoming internal conflicts, particularly by the third and second century BC, translated into success against external enemies, which in turn provided the material resources to diffuse internal tensions [95, 98]. However, increasing numbers of indebted small farmers nevertheless lost their farms, and though at first the propertyless *proletarii* were drawn to one of dozens of new colonies, after 180 BC only one new colony is reported [94, 99, 100]. The Crisis of the Republic, beginning in 134 BC, was triggered by attempts to reinstitute laws limiting interest rates and land concentration, leading to the end of the Republic in a century of civil war that, amongst other things, settled the debt question in favour of the wealthy creditors [78, 94, 101]. A little over a century later, the Roman Principate reached its largest territorial extent under the emperor Trajan in 117 AD, but his successors no longer pursued a policy of expansion and increasingly debased the coinage to cover expenditures. The Crisis of the Third Century saw the end of the classical period in civil wars, territorial losses, political assassinations, and the widespread return of debt peonage [94, 96].

Keen to avoid such recurring debt crises, the early Christian Fathers prohibited the charging of usury, particularly drawing on Old Testament texts describing Hebrew practices likely borrowed from the ancient Mesopotamians [78, 96, 102, 103]. Across Europe serfdom became widespread, but by the beginning of the second millennium Europe began to emerge from its prolonged slump, and learning, commerce, and money-lending began to revive [96, 104]. Developing classical thought, debates came to focus on how to distinguish legitimate interest from illegitimate usury. The Catholic Church gradually permitted interest to be contracted on a variety of loans, but most were at one time or another abused to disguise usurious charges [97, 105, 106]. From the fifteenth century, Protestant reformers argued that these prohibitions should be relaxed; in any case, with the ubiquity of interest-bearing money markets and the development of ever more complex financial contracts, by the eighteenth century the Catholic Church would effectively concede that the distinction between interest and usury was no longer within their capacity to legislate [97].

Beginning in western Europe, the modern period saw the gradual relaxation of laws restricting the kinds of contract that could be entered into for the transfer of resources. Among the earliest, England’s post-Reformation pro-creditor laws in 1543 and 1545 legislated for the imprisonment and dispossession of defaulters and effectively decriminalised all loan contracts that charged interest at less than ten per cent [107, 108]. As in classical times, the reinstitution of titled property institutions were accompanied by internal tensions mitigated by external expansion. English territories grew from the smallest they had been for hundreds of years through the complete recolonisation of Ireland by 1603, and in successive centuries the founding of colonies across the world from which resources were acquired and to which millions of people migrated or were transported; by 1925 the British empire covered almost a quarter of the Earth’s total land area [109, 110].

### The continuing evolution of ownership

Unlike earlier periods, the resource base of modern economies has continued expanding not only territorially but also through the use of vast reserves of fossil fuel energy, first from coal, then later increasing inputs of oil and natural gas [111, 112]. In England, by the mid-sixteenth century, rising demand for coal coincided with the localised depletion of woodland close to urban centres [113, 114]. Between 1550 and 1700, English coal output increased twelve-fold [115]. By the start of the nineteenth century coal-driven steam engines were sufficiently fuel efficient that they could for the first time be located away from a coal mine [116], including in countries where their use had previously been impossible, and to power the steam ships plying the long trade routes of the British Empire [117]. Globally, per capita energy consumption continues to increase [118], and unprecedentedly large urban populations are now sustained by food and fuel brought into cities using transports themselves powered largely by fossil fuels [119].

Since the late twentieth century, however, global energy growth has begun to slow [120]. The energy return from extracting fossil fuels has undergone a steady decline as higher quality resources have become depleted, leading to the exploitation of ever lower quality fuels that require ever more energy to obtain and refine [120]. Nor are nuclear fuels the panacea they once appeared, since the mining, enrichment, conversion, and disposal processes involved in nuclear technologies are themselves heavily dependent on fossil fuels and relatively scarce minerals for reactor construction. Moreover, the quality of available nuclear ore is itself declining, with high grade ore predicted to become rapidly depleted in the next few decades [120–122]. Renewable sources, though not susceptible to depletion, have their limits. Direct solar radiation is the only renewable resource that has the physical potential to surpass the amount of energy currently provided by fossil fuels, but the land that can be used to support photovoltaic generation without directly competing with food production is limited. Estimates suggest that unrealistically large amounts of land would be required for renewables to substitute for even 15 to 30 percent of current fuel use [116, 123].

Whilst titled property institutions have historically survived as long as resource transfers can be motivated by the promise of increased returns, in the absence of expansion the motivation to transfer resources through the promise of increased returns declines. The analysis presented here suggests that, in past societies, this has often led to social polarisation and a return to more directly coercive ownership forms. A better understanding of the evolution of our current ownership institutions seems increasingly important as our own society approaches ever more significant constraints [11, 79, 80].

## Discussion

### Situating the model within the existing literature

The model here differs from others in the literature in that it seeks to examine the evolution among human societies of communal, command, and titled property institutions, characterising these forms of ownership as institutions that govern the transfer of resource items. It therefore differs from the existing literature in six key ways.

First, I have here characterised communal, command, and titled property ownership institutions as rules governing the transfer of resource items. This characterisation therefore differs from an oft used typology within the social science literature that classifies private, public, common, and club ownership in terms of excludability and rivalry [124]. The conceptualisation of ownership as transfer rules, as used in this paper, therefore represents a complementary but distinct way of thinking about ownership, in terms of how items are either retained or transferred according to communal, command, or titled property rules.

Second, the evolutionary model here is constructed in contrast to an economic literature in which human ownership institutions are viewed as the result of when the marginal costs of asserting ownership are exceeded by the marginal benefits of reducing externalities, so that a mutual respect for the possessions of others reduces the costs of fighting required to defend those possessions [125, 126]. Though papers in this tradition often talk in terms of the ‘evolution’ of these institutions, [127–129] the term is used loosely to mean merely ‘change’ and there is little discussion of how processes of variation, selection, and retention might actually bring about the many different forms that ownership takes [130].

Third, the existing evolutionary game theory modelling literature has focussed on the variation, selection, and retention of behaviours as they evolved in cellular and chromosomal processes and nonhuman animals, and have not yet been extended to examine the ownership institutions of humans that are my focus here [2, 7, 131–134]. Moreover, these existing models aim to explain similarities across this wide range of processes independent of their different ecological circumstances [7]. In contrast, I am here examining the reasons for differences in the ownership institutions that govern different types of resource, and so I focus not on the similarities but on the different acquisition and retention strategies in different resource settings, providing a heuristic for examining how these different institutions have emerged and survived. Relatedly, unlike other economic and biological models [131, 135–137], the model in this paper is not intended as an account of the mechanisms by which some form of ownership might prevent a ‘tragedy of unmanaged commons’ from occurring. However, though the model here is itself silent upon the important question of whether ownership may limit the appropriation of items from a depletable resource stock, it is worth noting that the analysis that emerges does seem to suggest that the depletion of resources may occur under all four ownership institutions here modelled, to some extent reflecting empirical findings that ownership often fails to prevent resource depletion [138].

Fourth, given this focus on human institutions, the model in this paper builds upon the observation that under the communal, command, and titled property ownership characteristic of human societies, resource items are typically not retained by their first possessor but are transferred to others. This differs from Hawk-Dove-Bourgeois type models that aim to provide an account of the conventions observed in nonhumans by which the first possessor of a resource tends to retain possession of a resource. Variants include the Hawk-Dove-Bourgeois-Assessor model whereby disputes over possession are settled according to assessments about the rate of expenditure of a fitness budget during a fight [139], the ‘competitive collusion’ models in which the first individuals to arrive collude to prevent the establishment of further competitors [140], and the ‘nagging neighbour’ models in which territorial arrangements are determined through aggressive interactions [141]. However, it is oft remarked that Hawk-Dove-Bourgeois type models cannot explain the absence of an ‘anti-bourgeois’ convention in which existing possessors surrender their possessions to newcomers [7], and nor can such models explain the emergence of the non-possessive institutions typical of human groups. For clarity, the model presented here is not intended to downplay the role that prior possession plays in underpinning several human ownership institutions, for example where possession is used to stake an ownership claim to unowned resources as diverse as wild animals, abandoned treasure, and deep sea minerals [142]; in this regard, it is worth noting that the model preserves the way in which a prior possession convention continues to play a role in the emergence of the non-possessive institutional outcomes, since the benefits of retention or transfer can only accrue to those individuals who have made an effort to acquire an item in the first place. Nevertheless, among humans, most of the objects that people own have not been acquired by themselves, but by somebody else. The purpose of this paper has therefore been, not to downplay the role of prior possession rules, but to try to better understand the evolution of the rules that govern resource transfers.

Fifth, one further reason that both economic and Hawk-Dove-Bourgeois type models have tended to neglect the difference between animal possessiveness and the non-possessiveness characteristic of humans is that those literatures have largely focussed on the ownership of territory, rather than on the rules governing the ownership of resource items. With my focus on modelling resource transfers, I have distinguished ownership of territory from ownership of resource items, focussing on the ownership of resource items in order to better understand the rules governing their transfer and retention. Since my focus is on humans, this is an approach consistent with those within the anthropological tradition who focus less on the presence or absence of territorial boundaries, and more on the ownership rules that govern the ownership of resource items [143].

Sixth, the model allows the possibility that there may be a net benefit for an individual to transfer a resource item. This is in contrast to Producer-Scrounger models [144–146] which are constructed to examine the behaviour of group foragers by explaining the composition of a mixed population of Producers who try to obtain new resources and Scroungers who attempt to obtain existing resources from those Producers. As in the model here, Producer-Scrounger models focus on resource items and on the circumstances in which resources are transferred, and predict that a mix of Producers and Scroungers can be evolutionarily stable (similar to the Demander-Transferrer populations here), whilst a population composed of large numbers of Scroungers would likely force the group to dissolve (similar to the Demander dominated populations here). However, the focus of Producer-Scrounger models is still on explaining animal behaviours, specifically the scrounging patterns observed amongst social foragers such as primates and birds, and so though these models provide much more realistic and precise analyses of scrounging among social foragers than is possible in the more general model here, those models are not suitable for the examination of the evolution of ownership institutions among humans. Most specifically, producer-scrounger theories assume that scrounging reduces producer fitness, an assumption that has been noted as a possible limitation even in theorising social forager behaviour, since primate food-calling behaviour may increase the net fitness of the food-caller despite apparently encouraging scrounging [145]. Unlike Producer-Scrounger models, then, the model presented here explicitly allows resource transfers to increase the net fitness of agents, and thereby permits of the possibility that such benefits may play a role in evolution of human ownership institutions.

### Model limitations

Scientific models cannot simultaneously maximise generality, realism, and precision [143]. Since I use the model to examine a broad range of institutions, in this paper I have tended to privilege generality over realism, and to a lesser extent realism over precision. As a result, the model’s chief limitations are due to its simplicity, the high level of abstraction due to the use of ‘fitness’ as the variable unit measure, and the simplification of the passage of time.

The first limitation, that the model is simplistic, results from its focus on just five variables that place the analytical focus on intra-group interactions. As a result, the model neglects more complicated multi-level effects such as inter-group interactions like warfare, which are included in the model only to the extent that such activities impact upon the costs and benefits of interactions within the group, though group selection effects are widely thought to influence learned behaviours [12, 13]. Similarly, for simplicity, the model neglects policing [131] and the enforcement of policing [12] in populations of non-related individuals. Also neglected are costs and benefits of relatively small magnitude, such as the costs of moving between opportunities to play Demand [145], which are assumed to be negligible in comparison to the other variables included in the model.

The second limitation is that the model is abstract since it uses ‘fitness’ as the unit measure of the model variables. Standard practice in evolutionary modelling, such abstraction is very useful for producing a highly generalisable model, though it relegates much of the work of precision to the interpretative stage. In the case of the model presented here, four of the five variables are defined in terms of the increases or decreases they bring about in an individual’s fitness, and the fifth (*P*) is used only as a factor of one of the other four (*v*). The relationship between the variables is therefore expressed in terms of the relative values of fitness increases and decreases. This implies that resources are treated as inherently continuous, an assumption that is nevertheless reasonably realistic for my purposes, since the resources that are the focus are foraged foods, large game, agricultural yields, and fuels—all resources that are in principle indefinitely divisible. A related consequence of the use of an abstract ‘fitness’ unit as the model measure is that a given cost or benefit is defined in quite an aggregated manner. As such, it is not possible within the model to recreate the specific actions of individuals in particular circumstances, for which a finer-grained model would be required. As an illustrative example, the model cannot describe in detail the events that occur during a specific hunt should a number of individuals forego their foraging activities to pursue some larger prey, incurring costs in the pursuit and then attempting to obtain shares of the kill [27, 29, 30]. At the level of abstraction at which the model here operates, the best approximation of such events is one where, for a given increase in an individual’s fitness due to obtaining a resource through either their own or another’s kill, that individual must engage in activities which reduces their fitness by some proportional amount, with such activities taken to include all the unsuccessful hunts which they have joined. Despite this limitation of abstraction, in at least one important respect the model here nicely illustrates a key characteristic of human hunter-gatherer behaviour, since the distribution of meat in hunter-gatherer societies typically does not take place only amongst the individuals present at the kill, but amongst members of the community more widely. This is a distinguishing feature of communal ownership: that distribution occurs irrespective of any individual efforts made to join a hunt or to be in the vicinity of a kill.

The third broad limitation of the model is that the passage of time is modelled only in the transition from one generation of the population to the next, so all costs and benefits must be modelled as occurring as an instantaneous outcome from each interaction with payoffs that influence the population structure of the next generation of the game. Though it is intuitive to imagine that the costs and benefits of acquiring and of fighting to obtain or retain a resource are more or less immediate, the varied and diffuse benefits that may derive from transferring a resource are less intuitive to model as immediately occurring. Combined with the abstract nature of ‘fitness’, this means that some interpretative work is required to disambiguate the different ways in which fitness may be increased by transferring a resource of a particular value. Nevertheless, this modelling limitation is useful in illuminating the nature of the benefits that may be involved, since it highlights the problem that the benefits of transferring resources is not usually instantaneous in actual human societies. Broadly, the kinds of benefits to transferring are conceptualised here as those that derive from group membership, and though there may in reality be diverse costs and benefits involved in the addition of group members [147], for simplicity such benefits are here modelled as essentially costless to existing members. So, for example, a successful hunter who transfers food to others increases their own fitness by increasing the likelihood that other members of the group will survive, thereby increasing the extent to which a larger, better fed group will provide greater group defence and reduce variance in the future food supply. Though these benefits of transferring are diffuse and project into the future, it is worth noting that those who transfer resources under each of the ownership institutions often do immediately receive some form of assurance that they can expect some future benefits: hunters who share generously may gain prestige; peasants may be reassured that they will receive protection from expulsion and future attack; and parties to a contract are legally assured that they will be paid what they have contracted. Future work may usefully examine the similarities and differences between these different assurance mechanisms as they operate under these different institutions.

## Conclusion

In this paper, I have presented and interpreted a simple model for the continuing evolution of ownership among humans. Departing from existing models of possessive behaviours among nonhuman animals, the model provides a heuristic for examining the evolution of communal, command, and titled property institutions prevalent in human societies as institutions governing the transfer of resource items. Using the model to interpret existing evidence on the emergence and persistence of these forms of ownership suggests that each of these particular institutions is more likely to survive to govern particular types of resource. Specifically: communal ownership is adaptive among large game hunters once better hunting tools increase both the costs of fighting between group members and the defensive and variance reducing benefits of sustaining other members in the group; command ownership emerges after the decline of large game, when alternative resources become more easily monopolised by those with an increased ability to win conflicts and where outside conditions are such that even the lowliest member of society receives sufficient benefit upon the surrender of resources to those in command; and titled property becomes adaptive when written contracts provides a new means for individuals to ensure that they receive a benefit from making transfers. This analysis suggests that titled property institutions have historically declined when governing resources that are no longer expanding, and that such institutions may again come under pressure should future resources become significantly constrained.

## Method

### The Demander-Resister-Transferrer model

The three strategies can be expressed in terms of the five parameter variables as follows (see Table 2 for the list of symbols):

- *Demand* (D): demands a resource of value *v* from the other player. If the other player resists then there is a fight where D incurs a cost *f*. Demand wins the fight a proportion *P* of the time and gains *v* when it wins.
- *Resist* (R): Obtains a resource of value *v* at a cost *c*. Incurs a cost *f* if fought by a Demander; wins the fight and retains the resource 1 − *P* of the time.
- *Transfer* (T): Obtains a resource of value *v* at a cost *c*. Transfers the resource if demanded by a Demander with neither Transferrer nor Demander suffering any cost of fighting, and some additional benefit *b* may be gained by the Transferrer.

**Table 2:** List of symbols.

*D*	The Demander strategy.
*R*	The Resister strategy.
*T*	The Transferrer strategy.
*v*	Value of the resource obtained by the individual.
*c*	Cost of obtaining that resource from the environment; 0 < *c*.
*f*	Cost of conflict (‘fighting’) over the resource; 0 < *f*.
*b*	Benefit of transferring the resource.
*P*	Proportion of conflicts won by a Demander; 0 ≤ *P* ≤ 1.
*p*	Proportion of *D* in the population.
*q*	Proportion of *R* in the population.
1 − *p* − *q*	Proportion of *T* in the population.
*V*(*X*|*Y*)	The change in fitness of *X* when *X* interacts with *Y*.
*W*(*X*)	The average change in fitness across all of *X*’s interactions.

Different values for these five variables leads to different mixes of strategies in the population. These are represented by the proportion *p* in a population that adopts Demand, the proportion *q* that adopts Resist, and the proportion 1 − *p* − *q* that adopts Transfer. *V*(*X*|*Y*) denotes the increase in fitness that results from an interaction between some strategy *X* and some strategy *Y*, and *W*(*X*) denotes the average change in fitness across all of some strategy *X*’s interactions, which in turn depends upon the proportion of each of the other strategies in the population. The outcome of each interaction can therefore be listed as:

$$\begin{array}{ccc} V(D|D)=0 & V(R|D)=(1-P)v-f-c & V(T|D)=b-c\\ V(D|R)=Pv-f & V(R|R)=v-c & V(T|R)=v-c\\ V(D|T)=v & V(R|T)=v-c & V(T|T)=v-c \end{array}$$

and the average change in fitness across each of the three strategies listed as:

$$\begin{array}{cc} W(D) & =pV(D|D)+qV(D|R)+(1-p-q)(D|T)\\ s & =q(Pv-f)+(1-p-q)v \end{array}$$ (1)

$$\begin{array}{cc} W(R) & =pV(R|D)+qV(R|R)+(1-p-q)V(R|T)\\ & =v(1-pP)-pf-c \end{array}$$ (2)

$$\begin{array}{cc} W(T) & =pV(T|D)+qV(T|R)+(1-p-q)V(T|T)\\ & =(1-p)v+pb-c. \end{array}$$ (3)

The replicator dynamic is used to calculate the strategy mix in a population based upon the proportions in the preceding population and the relative fitness of the strategies. For the sake of argument, the model uses the standard replicator dynamic [14]. For a two strategy game between Demanders and Transferrers, for example, where *p*′ denotes the proportion of D in the succeeding population, this dynamic is:

$$\begin{array}{c} p^{\prime }=\frac{pV(D)}{pV(D)+(1-p)V(T)} \end{array}$$ (4)

from which the difference equation can be derived [14]:

$$\begin{array}{c} \Delta p=p\left(1-p\right)\frac{W(D)-W(T)}{pW(D)-(1-p)W(T)}. \end{array}$$ (5)

Strategies are evolutionarily stable when either one strategy dominates, or there is coexistence between two or more strategies. Within the model parameters of our model, only three outcomes are stable: domination by Demanders, domination by Resisters, or Demander-Transferrer coexistence.

Transferrers cannot dominate the other two strategies, since when they meet Resisters their payoffs are equal, and they would only always be fitter in their interactions with Demanders if both −*c* + *b* > 0 and *v* − *c* > *v*, yet the second of these cannot hold, since *c* > 0. Demanders can dominate: they will be fitter across all interactions with Resisters if 0 > (1 − *P*)*v* − *f* − *c* holds (as well as whenever 0 > (1 − *P*)*v* + *f* − *c* which, since *f* > 0, holds whenever the first inequality holds); they will also be fitter across all interactions with Transferrers if *c* > *b* (and whenever *v* > *v* − *c*, which always holds since *c* > 0). Correspondingly, Resisters can dominate Demanders when (1 − *P*)*v* − *f* − *c* > 0 (and when (1 − *P*)*v* + *f* − *c* > 0 which, since *f* > 0, holds whenever the first inequality holds), and in interactions with Transferrers will be equally fit since Resister and Transferrer payoffs are equal.

If 0 > (1 − *P*)*v* − *f* − *c* and *b* > *c* then no strategy will dominate, but a mixed population will result. In a coexisting Resister-Transferrer population the payoffs between the two strategies are equal, so its ability to repel Demanders depends upon the proportions of the mix, that is, whether there is a sufficient proportion of Resisters in the population to repel Demanders.

Demander-Transferrer coexistence is possible where *v* > *v* − *c* and *b* > *c* (but not the reverse, since *c* > 0). A Demander-Transferrer population can repel Resisters when both are fitter than invading Resisters. To find where this occurs, we first find the Demander-Transferrer equilibrium point $\hat{p}$ (remembering that *q* = 0):

$$\begin{array}{cc} W(D) & =W(T)\\ (1-\hat{p})v & =v\left(1-\hat{p}\right)+\hat{p}b-c\\ \frac{c}{b} & =\hat{p}. \end{array}$$ (6)

We then find the conditions where at this equilibrium point, *W*(*D*) = *W*(*T*) > *W*(*R*):

$$\begin{array}{cc} W(D) & >W(R)\\ (1-\hat{p})v & >v\left(1-\hat{p}P\right)-\hat{p}f-c\\ \frac{c}{v(1-P)-f} & >\hat{p}. \end{array}$$ (7)

Substituting $\hat{p}$ with $\frac{c}{b}$, simplifying, and solving for zero:

$$\begin{array}{cc} \frac{c}{v(1-P)-f} & >\frac{c}{b}\\ 0 & >(1-P)v-f-b. \end{array}$$ (8)

This inequality holds whenever 0 > (1 − *P*)*v* − *f* − *c* and *b* > *c*. So, when 0 > (1 − *P*)*v* − *f* − *c* and *b* > *c*, a Demander-Transferrer population coexists and repels Resisters.

A coexisting Demander-Resister population is not possible. Using the same process as above, the equilibrium point of a coexisting Demander-Resister population would be $\frac{q(Pv-f)-v+c}{-vP-f}=\hat{p}$, and that population could repel Transferrers if $p=\frac{q(Pv-f)-v+c}{-vP-f}$ and *W*(*D*) > *W*(*T*). However, estimating that inequality and substituting $\frac{q(Pv-f)-v+c}{-vP-f}$ for *p* simplifies to the inequality (1 − *P*)*v* − *f* − *b* > 0; since 0 > (1 − *P*)*v* − *f* − *c* and *c* > *b*, this outcome is not possible.
